# Associations of urinary caffeine metabolites with sex hormones: comparison of three statistical models

**DOI:** 10.3389/fnut.2024.1497483

**Published:** 2025-01-07

**Authors:** Jianli Zhou, Linyuan Qin

**Affiliations:** ^1^Department of Science and Education, Guilin People’s Hospital, Guilin, China; ^2^Department of Epidemiology and Health Statistics, School of Public Health, Guilin Medical University, Guilin, China; ^3^Guangxi Key Laboratory of Environmental Exposomics and Entire Lifecycle Health, Guilin, China

**Keywords:** Bayesian kernel machine regression, caffeine metabolites, multiple linear regression, sex hormone, weighted quantile sum regression

## Abstract

**Aims:**

The association between urinary caffeine and caffeine metabolites with sex hormones remains unclear. This study used three statistical models to explore the associations between urinary caffeine and its metabolites and sex hormones among adults.

**Methods:**

We selected the participants aged ≥18 years in the National Health and Nutrition Examination Survey (NHANES) data 2013–2014 as our study subjects. We performed principal components analysis (PCA) to investigate the underlying correlation structure of urinary caffeine and its metabolites. Then we used these principal components (PCs) as independent variables to conduct multiple linear regression analysis to explore the associations between caffeine metabolites and sex hormones (E2, TT, SHBG). We also fitted weighted quantile sum (WQS) regression, and Bayesian kernel machine regression (BKMR) methods to further assess these relationships.

**Results:**

In the PCA-multivariable linear regression, PC2 negatively correlates with E2: *β* = −0.01, *p*-value = 0.049 (male population). In the WQS regression model, the WQS indices were associated with SHBG and TT both in male (SHBG: WQS index = −0.11, *p* < 0.001; TT: WQS index = −0.10, *p* < 0.001) and female (SHBG: WQS index = −0.10, *p* < 0.001; TT: WQS index = −0.04, *p* < 0.001) groups. Besides, the WQS index was significantly associated with E2 in females (*p* < 0.05). In the BKMR model, despite no significant difference in the overall association between caffeine metabolites and the sex hormones (E2, TT, SHBG), there was nonetheless a declining trend in the male population E2 group, in the male and female population SHBG groups also observed a downward trend.

**Conclusion:**

When considering the results of these three models, the whole-body burden of caffeine metabolites, especially the caffeine metabolites in the PC2 metabolic pathway was significantly negatively associated with E2 in males. Considering the advantages and disadvantages of the three statistical models, we recommend applying diverse statistical methods and interpreting their results together.

## 1 Introduction

Sex hormones play crucial roles in regulating various physiological processes in the human body, and essential for reproductive function, bone health, cardiovascular health, and have a significant impact on metabolic processes (1–4). Estradiol (E2), sex hormone-binding globulin (SHBG), and total testosterone (TT) are three main sex hormones in the human body (5). Alterations in the levels of these hormones can lead to a range of health issues. E2 is a female hormone that is produced largely by the ovaries, but also by the adrenal glands, fat and liver, crucial for female reproductive health and is involved in the regulation of the menstrual cycle and pregnancy, levels of E2 can be associated with risk of breast cancer and uterine fibroids (6–8). SHBG is a protein produced by the liver that binds to sex hormones, regulating their transport and availability in the bloodstream, SHBG levels can also influence the availability of sex hormones, with high SHBG levels related to decreased risks of breast and prostate cancers (9, 10). TT is primarily produced in the testes and is essential for male sexual development and function. It plays a crucial role in the regulation of muscle mass, bone density, and libido, low levels of TT and E2 can result in reduced sexual function, decreased bone density, and an increased risk of cardiovascular disease (11–14). Therefore, understanding the factors that can influence the levels of these hormones is of utmost importance for maintaining overall health and preventing diseases.

In recent years, there has been growing interest in exploring the potential relationship between environmental factors and sex hormone levels. Caffeine, a widely consumed stimulant, has come under the spotlight due to its widespread use and potential endocrine-disrupting effects. Some studies suggested that caffeine may interfere with the synthesis or metabolism of sex hormones, leading to alterations in their levels (15–17), this raises concerns about the long-term impact of caffeine consumption on hormonal balance and health outcomes. For example, an animal study suggests that serum E2 levels were elevated in the caffeine-fed animals after 2 and 4 weeks of exposure (18). The research conclusions between caffeine and SHBG and TT are inconsistent. Some studies indicated that SHBG levels were positively associated with increasing caffeine intake (17, 19), while another study suggested that consumption of high caffeine was associated with a reduced risk of low SHBG (20). For TT, a study demonstrated that no linear association was identified between levels of caffeine intake and TT (21). However, another study demonstrated that compared with nondrinkers, participants who drank ≥4 cups of total coffee/d had higher concentrations of TT (15). In addition, an animal study suggested that there were lower levels of serum testosterone in the caffeine-fed group than the control group (16). Although there have been some studies exploring the relationships between sex hormones and caffeine intake. So far, few studies have investigated the relationships of sex hormones with caffeine and caffeine metabolites. More than 70% of caffeine *in vivo* comes from coffee consumption, and 70–80% of caffeine is formed from 7-dimethylxanthine (84%), theobromine (12%), and theophylline (4%) through 3-N demethylation reaction catalyzed by CYP1A2 enzyme (22–24). Based on the above inconsistent research of caffeine with E2, SHBG and TT, and given the metabolites of caffeine can also affect sex hormone levels, therefore, we sought to determine the associations of caffeine and its 14 caffeine metabolites with E2, SHBG and TT. We selected three statistical methods, including multiple linear regression, weighted quantile sum (WQS) regression, and Bayesian kernel machine regression (BKMR) models, to better analyze caffeine metabolites on E2, SHBG and TT. These three methods have their advantages and disadvantages, and we expected that this comprehensive analysis would yield insightful and fruitful conclusions.

## 2 Materials and methods

### 2.1 Study population

The National Health and Nutrition Examination Survey (NHANES) is a stratified, multi-stage survey of all non-institutionalized persons in the United States, conducted by the National Center for Health Statistics within the Centers for Disease Control and Prevention, represents the most comprehensive assessment of the health and nutritional status of both adults and children in the United States. It integrates data from interviews and physical examinations to provide a holistic view. In this study, we extracted data from the NHANES examination conducted between 2013 and 2014. Sociodemographic data, personal lifestyle data, as well as laboratory data of sex hormone concentrations and caffeine and its metabolites concentrations, have been included in our study for analysis. The exclusion criteria were as follows: (1) age < 18 years old; (2) ever use sex hormones; (3) positive laboratory pregnancy test or self-reported pregnancy at exam; (4) participants with missing E2, SHBG and TT values. The final study included 5550 participants (Supplementary Figure S7). Ethical approval was given by the National Center for Health Statistics Ethics Review Board and all survey participants provided informed written consent. The protocol number for NHANES 2013–2014 was continuation of protocol #2011–17.

### 2.2 Measurement of urinary caffeine and caffeine metabolites levels

Professional phlebotomists collected 24-h urine samples from the participants. These specimens were stored in a frozen state, with temperatures maintained at –20°C for short-term storage and –70°C for long-term preservation. Additionally, they were kept in conditions that avoided exposure to light until they were transported to the National Center for Environmental Health for further testing. The utilization of high-performance liquid chromatography-electrospray ionization-tandem quadrupole mass spectrometry (HPLC-ESI-MS/MS) with stable isotope-labeled internal standards allowed for the quantification of urine caffeine and 14 of its metabolite levels. If the biomarker’s concentration was below the detection limit, it was given a value equal to the detection limit divided by $\sqrt{2}$ , the limit of detection of each caffeine or caffeine metabolite was shown in Supplementary Table S1. More details of laboratory methodology were described at: caffeine and caffeine metabolites–urine lab procedure manual: https://wwwn.cdc.gov/nchs/data/nhanes/public/2013/labmethods/CAFE-H-MET-508.pdf. 1-methyluric acid (URXMU1), 3-methyluric acid (URXMU2), 7-methyluric acid (URXMU3), 1,3-dimethyluric acid (URXMU4), 1,7-dimethyluric acid (URXMU5), 3,7-dimethyluric acid (URXMU6), 1,3,7-trimethyluric acid (URXMU7), 1-methylxanthine (URXMX1), 3-methylxanthine (URXMX2), 7-methylxanthine (URXMX3), theophylline (1,3-dimethylxanthine, URXMX4), paraxanthine (1,7-dimethylxanthine, URXMX5), theobromine (3,7-dimethylxanthine, URXMX6), caffeine (1,3,7-trimethylxanthine, URXMX7), and 5-acetylamino-6-amino-3-methyluracil (URXAMU), a total of 15 metabolites were measured.

### 2.3 Covariates

Based on previous studies, other variables which may affect sex hormone concentrations were included in our study as well (25). In detail, regular covariates such as sociodemographic variables including gender, age, race (Mexican American, Other Hispanic, Non-Hispanic White people, Non-Hispanic Black people, Other Race - Including Multi-Racial), education level (less than 9th grade, 9-11th grade, high school graduate/GED or equivalent, some college or AA degree, college graduate or above), poverty income ratio, marital status (married, widowed, divorced, separated, never married, living with partner). Moreover, health-related behavioral factors are as follows: smoking cigarettes (26) (whether at least 100 cigarettes in life or not), alcohol drinking (27) (whether drunk alcohol over the past 12 months), physical activity (28) (whether have vigorous work activity or moderate work activity or not), body mass index (BMI) (29), estimated glomerular filtration rate (eGFR) (30) and daily sleep time (31).

### 2.4 Sex hormones measurement

Serum samples are processed, stored, and shipped to the Division of Laboratory Sciences, National Center for Environmental Health, Centers for Disease Control and Prevention, Atlanta, GA for analysis. The measurements of E2 and TT in serum are performed using the isotope dilution liquid chromatography tandem mass spectrometry (ID-LC–MS/MS) method developed by the Centers for Disease Control and Prevention (CDC). This method was designed for high sample throughput and has demonstrated a high level of accuracy and precision over multiple years. SHBG is based on the reaction of SHBG with immuno-antibodies and chemo-luminescence measurements of the reaction products that occur after two incubation periods and subjected to a magnetic field. The microparticles are captured on an electrode, where a chemiluminescent reaction occurs and can be measured by a photomultiplier tube. The readings are compared to an instrument-and lot-specific calibration curve. Analytes with analytic results below the lower limit of detection were calculated as lower limit of detection divided by the square root of 2. The lower limit of detection for E2, SHBG, and TT are: 2.994 pg./ML; 0.800 nmol/L; and 0.75 ng/mL. For detailed information on the measurement procedures, please refer to the laboratory procedure manual available on the NHANES website.

### 2.5 Statistical analysis

In the analysis of non-normal continuous data, we utilized median and quartiles to characterize the central tendency and dispersion. For categorical data, frequency and percentage were adopted to quantify distribution patterns and relative importance. In this study, we employed PCA linear regression as the preliminary analysis to identify influencing factors; we used WQS regression analysis to investigate the overall mixed effects of caffeine metabolites and other factors on sex hormones, and explored the magnitude of influence of every factor; we applied BKMR ro explore the complex, potentially nonlinear, and non-additive relationship between caffeine metabolites and sex hormones.

#### 2.5.1 Statistical model1: PCA-multiple linear regression

As an exploratory technique, principal component analysis (PCA) was employed to investigate the potential correlational structure among 15 features. Orthogonal varimax rotation was applied to the factors, and principal components (PCs) with eigenvalues above 1 were retained. PCA effectively transformed highly correlated caffeine metabolites into a reduced set of uncorrelated PCs, minimizing inter-component correlations. Consequently, the use of PCs as independent variables mitigated multicollinearity among caffeine metabolites. For each retained PC, individual scores were calculated as linear combinations of caffeine and its metabolites weighted by their respective loadings. Subsequently, these PCs were used as predictors in multivariate linear regression analyses, with E2, TT, and SHBG as dependent variables.

We split the data into two parts based on gender, and fit linear regression models for each part separately. We establish these models by taking E2, SHBG, and TT as the dependent variables respectively, and incorporating PC1, PC2, and covariates such as age, race, education level, poverty income ratio, marital status, smoking and drinking status, physical activity, BMI, eGFR, and daily sleep time as the independent variables into the equations.

#### 2.5.2 Statistical model 2: WQS regression model

We applied a “mixtures” approach based on WQS regression models to explore the combined effects of caffeine metabolite exposure and all covariates included in this study. In WQS regression, a weighted index that represents the correlated caffeine metabolites mixtures and all covariates included in this study were constructed based on the quantiles of caffeine metabolites components, and then the outcome variable is regressed on this index with an assumption that all components of the index work in the same direction. In each WQS regression model, weights of the caffeine metabolites were calculated to identify the component that contributes to the main effect in the mixtures on sex hormones. The weights ranged from 0 to 1 and were summed to 1 to facilitate comparison. The dataset used in WQS regression was split into training and validation sets (40:60), where the training dataset was used to estimate variable weights, and the validation dataset was used to test mixture significance.

#### 2.5.3 Statistical model 3: BKMR model

We employed the BKMR model to assess the combined effects of caffeine and caffeine metabolites on sex hormones. This model integrates Bayesian and statistical learning methods and enables the identification of nonlinear and non-additive relationships. Due to the high correlation among metabolites, we implemented a hierarchical variable selection method using a Markov chain Monte Carlo algorithm 10,000 iterations. In addition to PCA-based groupings, we also fitted a model encompassing all caffeine metabolites.

Since covariates and predictor variables are linear influencing factors in linear regression, covariates can also be treated as predictor variables. When employing WQS regression to examine the impact of caffeine metabolites on sex hormones, we aimed to test and demonstrate the combined effects of these metabolites alongside other covariates, highlighting the magnitude of the effect of caffeine metabolites on sex hormones among various influencing factors. However, in BKMR, our sole objective was to explore the complex, potentially nonlinear, and non-additive relationship between caffeine metabolites and sex hormones. Therefore, factors such as age and gender were included in the equation as covariates with linear relationships.

In conducting the NHANES survey, a weighting procedure was employed to diminish the impact of selection bias among various subgroups, including those based on age, gender, and ethnicity. Nevertheless, for our analysis, we utilized unweighted estimations. This decision was made because the variables that were used to determine the sample weights had already been comprehensively included in the adjusted model, as suggested in previous recommendations (32).

We considered that gender may have an impact on the concentration of sex hormones, so we conducted gender subgroup analysis in multiple linear regression and WQS regression analysis. All statistical analyses were performed with R version R-4.3.3 and SPSS version 26 with a two-sided *p* value <0.05 considered as statistically significant.

## 3 Results

### 3.1 Demographic characteristics

The study comprises 52.5% males and 47.5% females (Table 1). Participants spanned diverse racial/ethnic backgrounds, including Mexican Americans (14.5%), Other Hispanics (9.1%), Non-Hispanic White people (40.1%), Non-Hispanic Black people (21.0%), and Other Races (15.3%). Education levels ranged from less than 9th grade (8.4%) to college graduates or above (24.9%). About marital status, with 51.3% were married. Smoking history was reported by 42.4% of participants, and 38.5% reported moderate or vigorous physical activity. The median age was 45 years, with a wide range of income-poverty ratios (1.97 [1.02, 3.92]). The median levels of urinary URXMU1, URXMU2, URXMU3, URXMU4, URXMU5, URXMU6, URXMU7, URXMX1, URXMX2, URXMX3, URXMX4, URXMX5, URXMX6, URXMX7, and URXAMU were 57.15, 0.48, 12.30, 6.08, 24.65, 0.81, 1.36, 26.60, 24.70, 39.90, 1.74, 17.10, 15.50, 3.59, and 57.85 umol/L, respectively. The median levels of E2, TT and SHBG were 24.40 pg./mL, 46.05 nmol/L, 182 ng/dL, respectively.

**Table 1:** Characteristics of participants included in the study.

Characteristics	*N* (%) or median (P25 P75)
Categorical variables	*N* (%)
Gender	
Male	2,916 (52.5)
Female	2,634 (47.5)
Race	
Mexican American	805 (14.5)
Other Hispanic	504 (9.1)
Non-Hispanic White people	2,226 (40.1)
Non-Hispanic Black people	1,168 (21.0)
Other Race–Including Multi-Racial	847 (15.3)
Education level	
Less than 9th grade	436 (8.4)
9–11th grade (Includes 12th grade with no diploma)	737 (14.2)
High school graduate/GED or equivalent	1,182 (22.7)
Some college or AA degree	1,552 (29.9)
College graduate or above	1,292 (24.9)
Marital status	
Married	2,667 (51.3)
Widowed	344 (6.6)
Divorced	565 (10.9)
Separated	166 (3.2)
Never married	1,071 (20.6)
Living with partner	390 (7.5)
Smoked at least 100 cigarettes in life	
Yes	2,353 (42.4)
No	3,196 (57.6)
Moderate or vigorous physical activity	
Yes	2,133 (38.5)
No	3,414 (61.5)
Continuous variables	Median (P25 P75)
Age (years)	45.00 [31.00, 61.00]
Income-poverty ratio	1.97 [1.02, 3.92]
Drinking (times/year)	2.00 [1.00, 4.00]
BMI	27.62 [23.84, 32.26]
eGFR (median [IQR])	99.31 [82.58, 113.27]
Sleep Duration (hours)	7.00 [6.00, 8.00]
URXMU1 (umol/L)	57.15 [23.23, 125.50]
URXMU2 (umol/L)	0.48 [0.16, 1.21]
URXMU3 (umol/L)	12.30 [4.39, 28.80]
URXMU4 (umol/L)	6.08 [2.29, 14.50]
URXMU5 (umol/L)	24.65 [7.12, 57.37]
URXMU6 (umol/L)	0.81 [0.30, 1.95]
URXMU7(umol/L)	1.36 [0.33, 3.79]
URXMX1 (umol/L)	26.60 [8.91, 65.00]
URXMX2 (umol/L)	24.70 [10.30, 55.02]
URXMX3 (umol/L)	39.90 [16.58, 92.05]
URXMX4 (umol/L)	1.74 [0.55, 3.62]
URXMX5 (umol/L)	17.10 [5.19, 36.82]
URXMX6 (umol/L)	15.50 [6.28, 33.70]
URXMX7 (umol/L)	3.59 [0.79, 9.50]
URXAMU (umol/L)	57.85 [19.50, 126.00]
E2 (pg/mL)	24.40 [15.20, 38.50]
SHBG (nmol/L)	46.05 [31.01, 69.65]
TT (ng/dL)	182.00 [21.40, 402.85]

URXMU1, 1-methyluric acid; URXMU2, 3-methyluric acid; URXMU3, 7-methyluric acid; URXMU4, 1,3-dimethyluric acid; URXMU5, 1,7-dimethyluric acid; URXMU6, 3,7-dimethyluric acid; URXMU7, 1,3,7-trimethyluric acid; URXMX1, 1-methylxanthine; URXMX2, 3-methylxanthine; URXMX3, 7-methylxanthine; URXMX4, 1,3-dimethylxanthine, theophylline; URXMX5, 1,7-dimethylxanthine, paraxanthine; URXMX6, 3,7-dimethylxanthine, theobromine; URXMX7, 1,3,7-trimethylxanthine, caffeine; URXAMU, 5-acetylamino-6-amino-3-methyluracil.

### 3.2 Principal component of caffeine and caffeine metabolites

The main PCs derived from the PCA are summarized in Table 2. Notably, the first two components had eigenvalues ≥1, cumulatively explaining 76.71% of the total variance. Table 3 presents the rotated component matrix of urinary caffeine and its 14 metabolites, focusing on those exhibiting correlations greater than 0.5. Specifically, the first component (PC1) exhibits robust correlations with URXAMU, URXMU1, URXMU4, URXMU5, URXMU7, URXMX1, URXMX4, URXMX5 and URXMX7. The second component (PC2) correlated with URXMU2, URXMU3, URXMU6, URXMX2, URXMX3, and URXMX6.

**Table 2:** Total variance showed by components of principal component analysis.

Component		Initial eigenvalues	
	Total	Proportion of variance (%)	Cumulative proportion (%)
1	7.48	49.85	49.85
2	4.03	26.86	76.71
3	0.95	6.36	83.07
4	0.72	4.82	87.89
5	0.53	3.50	91.39
6	0.36	2.39	93.78
7	0.25	1.63	95.41
8	0.17	1.10	96.51
9	0.14	0.96	97.47
10	0.12	0.80	98.27
11	0.09	0.57	98.84
12	0.08	0.51	99.35
13	0.04	0.28	99.63
14	0.03	0.20	99.82
15	0.03	0.18	100.00

**Table 3:** Rotated component matrix of 15 urinary caffeine and caffeine metabolites.

Caffeine metabolites	PC1	PC2
URXAMU (umol/L)	0.795	
URXMU1 (umol/L)	0.835	
URXMU2 (umol/L)		0.847
URXMU3 (umol/L)		0.952
URXMU4 (umol/L)	0.779	
URXMU5 (umol/L)	0.916	
URXMU6 (umol/L)		0.933
URXMU7(umol/L)	0.869	
URXMX1 (umol/L)	0.815	
URXMX2 (umol/L)		0.955
URXMX3 (umol/L)		0.952
URXMX4 (umol/L)	0.805	
URXMX5 (umol/L)	0.846	
URXMX6 (umol/L)		0.839
URXMX7 (umol/L)	0.781	

Factors with coefficients (or factor loadings) whose absolute values were less than 0.5 were not displayed and can be considered as having insignificant correlations. URXMU1, 1-methyluric acid; URXMU2, 3-methyluric acid; URXMU3, 7-methyluric acid; URXMU4, 1,3-dimethyluric acid; URXMU5, 1,7-dimethyluric acid; URXMU6, 3,7-dimethyluric acid; URXMU7, 1,3,7-trimethyluric acid; URXMX1, 1-methylxanthine; URXMX2, 3-methylxanthine; URXMX3, 7-methylxanthine; URXMX4, 1,3-dimethylxanthine, theophylline; URXMX5, 1,7-dimethylxanthine, paraxanthine; URXMX6, 3,7-dimethylxanthine, theobromine; URXMX7, 1,3,7-trimethylxanthine, caffeine; URXAMU, 5-acetylamino-6-amino-3-methyluracil.

### 3.3 Multiple linear regression analysis of urinary caffeine metabolite PC scores with E2, SHBG, and TT

We used PC1 and PC2 as independent variables and investigated their associations with sex hormones (Table 4). In the male population, negatively correlates with PC2 and E2: *β* = −0.01, *p*-value = 0.049, and there were no statistical associations between PC1 and E2, and when the dependent variables are SHBG and TT, there is no significance with PC1 and PC2. In the female group, we found that PC1 and PC2 were not statistically significant with E2, SHBG and TT (all *p* values were greater than 0.05).

**Table 4:** Multiple linear regression analysis of PC scores of urinary caffeine and caffeine metabolites with E2, SHBG and TT.

Population	Variables	*β* (95% CI)	*p*-value
Male	E2		
	PC 1	0.00 (−0.01,0.01)	0.805
	PC 2	−0.01 (−0.03,0.00)	**0.049**
	SHBG		
	PC 1	−0.01 (−0.02,0.01)	0.379
	PC 2	0.00 (−0.03,0.02)	0.678
	TT		
	PC 1	−0.01 (−0.03,0)	0.076
	PC 2	−0.01 (−0.03,0.01)	0.239
Female	E2		
	PC 1	0.03 (−0.02,0.08)	0.245
	PC 2	0.01 (−0.04,0.07)	0.632
	SHBG		
	PC 1	0.01 (−0.01,0.04)	0.363
	PC 2	−0.01 (−0.04,0.03)	0.727
	TT		
	PC 1	0.00 (−0.02,0.02)	0.721
	PC 2	0.00 (−0.03,0.02)	0.956

Models were adjusted for gender, age, race, education level, poverty income ratio, marital status, smoking and drinking status, physical activity, BMI, eGFR and daily sleep time. Bold value means *P*-value is less than 0.05.

### 3.4 WQS regression analysis of urinary caffeine metabolite levels with E2, SHGB, and TT

In males, the WQS indices were statistically associated with SHBG (WQS index = −0.11, 95%CI: −0.13 ~ −0.08, *p* < 0.001) and TT (WQS index = −0.10, 95%CI: −0.12 ~ −0.08, *p* < 0.001). For the female group, the WQS regression indices were statistically associated with E2 (WQS index =0.34, 95%CI: 0.25 ~ 0.44, *p* < 0.001), SHBG (WQS index = −0.10, 95%CI: −0.13 ~ −0.07, *p* < 0.001) and TT (WQS index = −0.04, 95%CI: −0.06 ~ −0.02, *p* < 0.001). Detailed results are presented in Table 5.

**Table 5:** Association between WQS index and E2, SHBG and TT.

Variables	WQS index (95% CI)	*p*-value
Male		
E2	−0.03 (−0.05,0)	0.056
SHBG	−0.11 (−0.13,-0.08)	<0.001
TT	−0.10 (−0.12, −0.08)	<0.001
Female		
E2	0.34 (0.25,0.44)	<0.001
SHBG	−0.10 (−0.13, −0.07)	<0.001
TT	−0.04 (−0.06, −0.02)	<0.001

**Figure 1:**
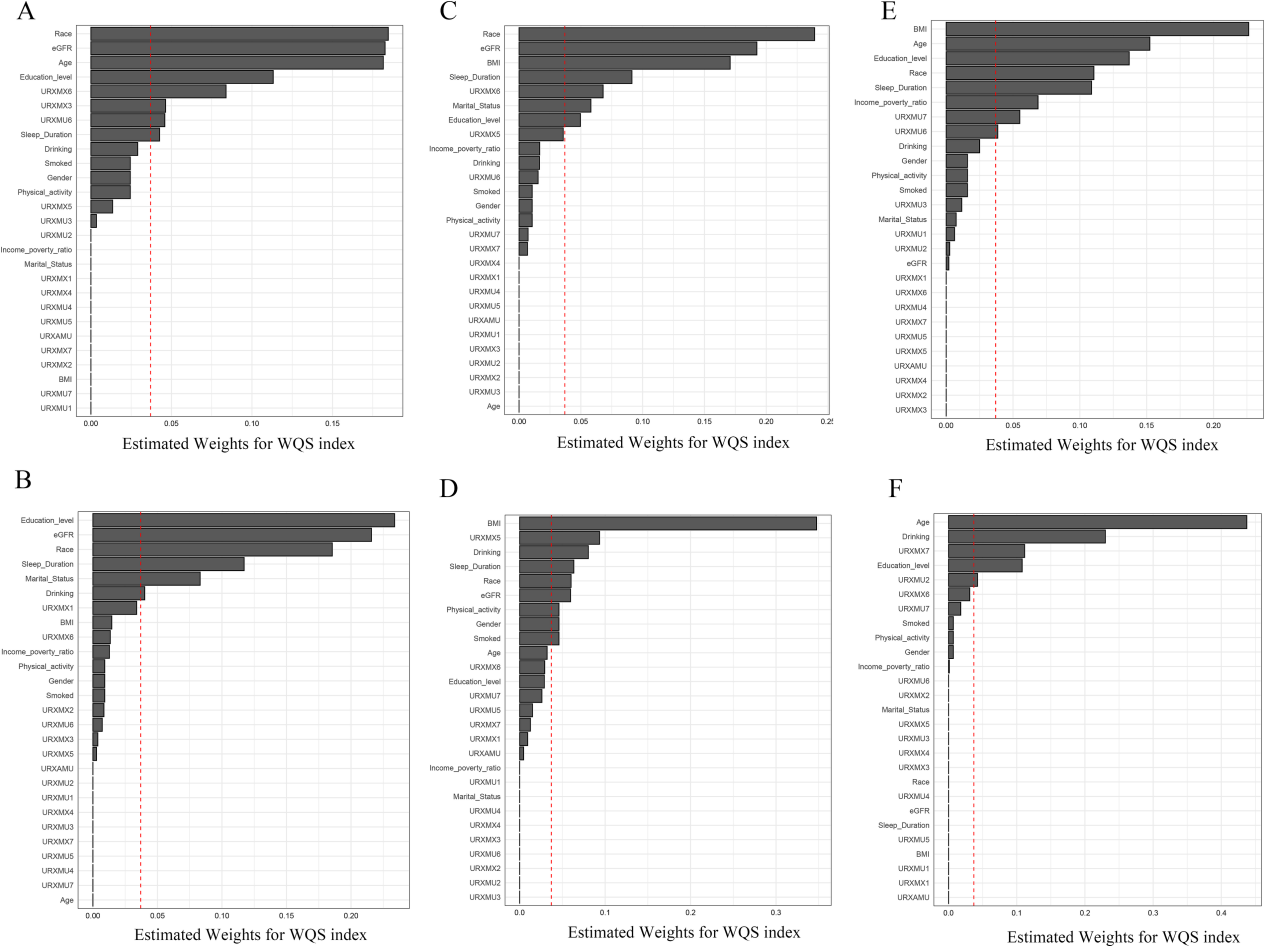
WQS model regression index weights for E2 **(A,B)**, SHBG **(C,D),** and TT **(E,F)** in both genders. **(A,C,E)**: Male; **(B,D,F)**: female. The red dashed line represents the reference for weights (1/number of components), and exceeding this reference implies that the component has a relatively large contribution to the overall effect on sex hormones among all the included influencing factors.

The estimated chemical weight for each WQS index is shown in Figure 1. In the male E2 model (Figure 1A), the weights of URXMX6, URXMX3, and URXMU6 exceeded the threshold line, while in the female E2 model (Figure 1B), none of the caffeine metabolites weight was identified as significant. In the male SHBG model, the weight of URXMX6 exceeded the significant line (Figure 1C), and in the female SHBG model, the weight of URXMX5 was significant (Figure 1D). In the male TT model, the weights of URXMU7 and URXMU6 were significant (Figure 1E), similarly in the female TT model, the weights of URXMX7 and URXMU2 were significant (Figure 1F).

### 3.5 BKMR model to assess the associations between caffeine metabolites and E2, SHBG and TT

We treated the concentration of each caffeine metabolites as continuous variables and categorized the caffeine metabolites into two non-overlapping groups based on PCA results (PC1 as group 1 and PC2 as group 2) to fit the BKMR model to assess the joint effect of exposures on continuous outcomes (log10-transformed E2、log10-transformed SHBG and log10-transformed TT). The probabilities of inclusion derived from the BKMR model for the two groups (groupPIP) and each metabolites (condPIP) are summarized in Table 6. In the male population E2 group, the groupPIPs of caffeine metabolites were all higher than 0.5. In addition, the condPIP of URXMU2 and URXMU6 were relatively high at 0.346 within this group, whereas the condPIPs of others in this group were low. While in the female population E2 group, only groupPIP of group 2 was higher than 0.5, and the condPIP of the URXMX6 was 0.538, relatively higher than other metabolites within this group. In the male population SHBG group, the groupPIPs of group 2 were higher than 0.5, the condPIP of URXMX3 and URXMX6 were relatively high at 0.333 and 0.300 within this group, whereas the condPIPs of others in both groups were relatively low. In the female population SHBG group, groupPIPs of group 1 were higher than 0.5, and the condPIP of URXMX4 was relatively high at 0.429. In the male population TT group, the groupPIPs of caffeine metabolites were all higher than 0.5, the condPIP of URXMU4 was extremely high at 1.000 within group 1, and URXMU6 was relatively high at 0.407 within group 2, whereas the condPIPs of others in both groups were low. In the female TT group, groupPIPs of group 2 were higher than 0.5, and the condPIP of URXMX3 was relatively high at 0.448.

**Table 6:** Posterior inclusion probabilities (PIPs) for group inclusion and conditional inclusion into E2, SHBG and TT models, using Bayesian kernel machine regression (BKMR) model.

Caffeine metabolites	Group	Male population E2		Female population E2		Male population SHBG		Female population SHBG		Male population TT		Female population TT	
		groupPIP	condPIP	groupPIP	condPIP	groupPIP	condPIP	groupPIP	condPIP	groupPIP	condPIP	groupPIP	condPIP
URXMU1	1	0.580	0.172	0.240	0.500	0.440	0.000	0.560	0.036	1.000	0.000	0.480	0.000
URXMU2	2	0.520	0.346	0.520	0.038	0.600	0.000	0.380	0.053	0.540	0.259	0.580	0.345
URXMU3	2	0.520	0.038	0.520	0.038	0.600	0.267	0.380	0.474	0.540	0.000	0.580	0.207
URXMU4	1	0.580	0.000	0.240	0.000	0.440	0.091	0.560	0.071	1.000	1.000	0.480	0.000
URXMU5	1	0.580	0.000	0.240	0.083	0.440	0.000	0.560	0.000	1.000	0.000	0.480	0.000
URXMU6	2	0.520	0.346	0.520	0.192	0.600	0.100	0.380	0.158	0.540	0.407	0.580	0.000
URXMU7	1	0.580	0.207	0.240	0.000	0.440	0.636	0.560	0.000	1.000	0.000	0.480	0.250
URXMX1	1	0.580	0.069	0.240	0.000	0.440	0.000	0.560	0.179	1.000	0.000	0.480	0.292
URXMX2	2	0.520	0.154	0.520	0.154	0.600	0.000	0.380	0.105	0.540	0.111	0.580	0.000
URXMX3	2	0.520	0.077	0.520	0.038	0.600	0.333	0.380	0.000	0.540	0.185	0.580	0.448
URXMX4	1	0.580	0.034	0.240	0.083	0.440	0.273	0.560	0.429	1.000	0.000	0.480	0.000
URXMX5	1	0.580	0.069	0.240	0.167	0.440	0.000	0.560	0.000	1.000	0.000	0.480	0.000
URXMX6	2	0.520	0.038	0.520	0.538	0.600	0.300	0.380	0.211	0.540	0.037	0.580	0.000
URXMX7	1	0.580	0.276	0.240	0.000	0.440	0.000	0.560	0.179	1.000	0.000	0.480	0.000
URXAMU	1	0.580	0.172	0.240	0.167	0.440	0.000	0.560	0.107	1.000	0.000	0.480	0.458

Models were adjusted for gender, age, race, education level, poverty income ratio, marital status, smoking and drinking status, physical activity, BMI, eGFR and daily sleep time.

**Figure 2:**
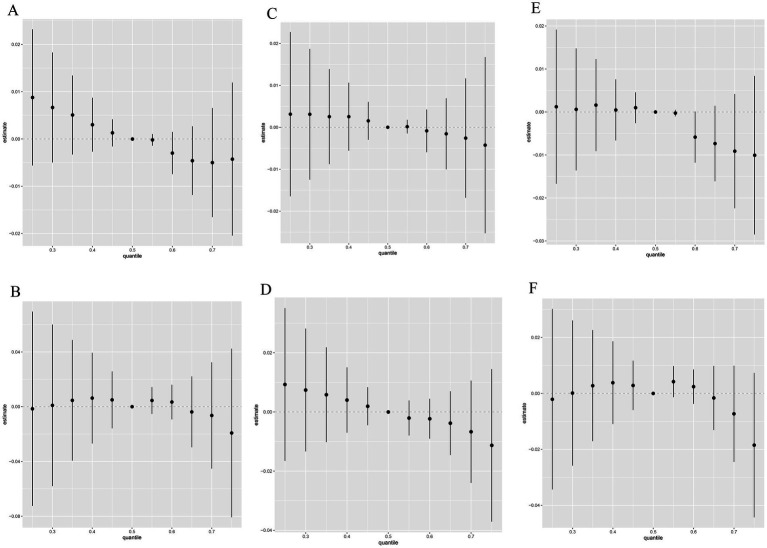
Relative effect of caffeine metabolites on E2 **(A,B)**, SHBG **(C,D)**, and TT **(E,F)** when comparing all the chemicals at different percentiles with their median level. **(A,C,E)**: Male; **(B,D,F)**: female. Models were adjusted for gender, age, race, education level, poverty income ratio, marital status, smoking and drinking status, physical activity, BMI, eGFR and daily sleep time.

The overall associations between the caffeine and caffeine metabolites mixture and the latent continuous outcomes are shown in Figure 2. Although no statistically significant differences were found in all the E2, SHBG and TT models, we still observed the curves between males and females were slightly different. In the male population E2 group, a declining trend was observed, while a flat curve in the female population was observed in Figures 2A,B. Both in the male and female population SHBG group, declining trends were observed and the slope of the female was larger Figures 2C,D. The trends of the male and female population TT group were different (Figures 2E,F).

The association of exposure-response functions of the fifteen caffeine and caffeine metabolites are shown in Supplementary Figures S1–S6. When all the other chemicals were at their median levels, URXMU6, URXMU7, and URXMX5 showed negative associations and URXMU1, URXMX2, and URXMX7 showed positive associations with E2 in male (Supplementary Figure S1); URXMU1 and URXMX6 showed the positive associations and URXAMU showed the negative association with E2 in female (Supplementary Figure S2). URXMU3 showed the positive associations and URXMU7 showed the negative association with SHBG in male (Supplementary Figure S3); URXMX4 showed the negative association with SHBG in females (Supplementary Figure S4). URXMU4 showed the negative association with TT in males (Supplementary Figure S5), URXMU7 and URXAMU showed the negative association and URXMX1 showed the positive association with TT in females (Supplementary Figure S6), although these relationships are not statistically significant.

## 4 Discussion

Caffeine, a xanthine alkaloid primarily found in beverages such as coffee, tea, cola, and dark chocolate, as well as certain analgesics (33, 34), has gained global popularity due to its efficacy in mitigating fatigue, energizing the mind, and enhancing stomach and heart function (35). Widely regarded as a psychoactive substance, caffeine is frequently employed as a stimulant for the central nervous system (36). Nevertheless, recent research and epidemiological evidence have increasingly highlighted its significant role in sex hormone metabolism as well (21, 37). In this study, we considered the results of diverse statistical methods to examine the effects of urinary caffeine and 14 caffeine metabolites on E2, SHBG and TT, among adults using data from NHANES 2013–2014. On one hand, the PCA-multiple linear regression pointed out the association between PC scores of urinary caffeine and sex hormones (E2, SHBG and TT). On the other hand, the WQS model identified the roles of caffeine and 14 caffeine metabolites in the development of E2, SHBG and TT. BKMR models were used to predict complex relationships between urinary caffeine and 14 caffeine metabolites and E2, SHBG and TT. This study emphasizes the significance of evaluating the combined impacts of chemicals on health outcomes by adopting diverse statistical approaches and contrasting their findings, while carefully weighing the benefits and drawbacks of each specific method.

In our study, multiple linear regression results showed that PC2 negatively correlates with E2 in males. While PC1 and PC2 were not statistically significantly associated with SHBG and TT both in male and female populations. A previous study found that higher coffee intake was associated with lower luteal total E2, this is consistent with the direction of our research findings, but not significantly associated with SHBG (38), while another study did not find any association between coffee intake and E2 (39). Previous studies with multiple linear regression analyses found associations between caffeine metabolites and TT, and the research conclusions were inconsistent, Lopez et al. found no association between caffeine intake and TT (21) but Glover et al. and Schliep et al. found that there was an inverse association between caffeine and TT (37, 40). We speculate that the reasons for the inconsistency between our study and previous research are as follows: firstly, the independent variables differ among studies. For instance, in the studies by Lopez et al. (21) and Schliep et al. (40), dietary caffeine intake was used as the independent variable, and investigating dietary caffeine intake may be subject to recall bias. Whereas in our study, urinary caffeine concentration served as the independent variable. Secondly, the statistical methods employed vary. For example, we conducted a principal component analysis (PCA) on 15 caffeine and its metabolites and used the PCA scores as the independent variable to explore their relationship with sex hormones. In contrast, in the study by Glover et al. (37), caffeine and its metabolites were used as independent variables and employed linear regression to investigate the relationship between caffeine and sex hormones. Multiple linear regression offers simplicity, interpretability, and a strong theoretical foundation for modeling the relationship between a dependent variable and one or more independent variables (41–43). By applying PCA, the method effectively converted the highly correlated caffeine metabolites into a condensed set of uncorrelated principal components (PCs), thereby minimizing the correlations among the different components. Subsequently, we employed these PCs as independent variables effectively addressed the multicollinearity among the original caffeine metabolites (44), this makes the results of our study more convincing.

WQS and BKMR models aimed at elucidating the health impacts of chemical mixtures, considering a series of highly correlated chemicals. Both models exhibit distinct strengths and limitations. The WQS regression model, specifically, focuses on quantifying the cumulative burden of chemical exposures throughout the body. This is accomplished by leveraging weights empirically derived through a bootstrap sampling process, providing a more holistic view of the intricate and multifaceted exposures encountered in real-life scenarios. This methodology effectively captures the complexities of real-world exposure patterns, making it a valuable tool for assessing the overall impact of chemical mixtures on health. In our analysis, the WQS indices of caffeine mixtures were significantly negatively associated with both SHBG and TT in both males and females, while significantly positively associated with E2 only in females. For figures of WQS model regression index weights, URXMX6, URXMX3 and URXMU6 were weighted highly in E2 (male population); URXMX6 (male population) and URXMX5 (female population) were weighted highly in SHBG. URXMU7 and URXMU6 were weighted highly in TT with the male population; URXMX7 and URXMU2 were weighted highly in TT with the female population. In the multiple linear regression results, we only found a negative correlation between PC2 and E2 in the male population, while in the WQS regression model, we found that some caffeine metabolites of PC1 and PC2 were related to SHBG and TT. Our research findings support previous studies, which have indicated that the WQS regression model is more sensitive than single-chemical analyses in identifying important factors (45, 46).

BKMR analysis and WQS regression differ significantly in their capabilities. BKMR excels in identifying nonlinear effects and intricate interactions among multiple chemicals, providing a more comprehensive view of exposure-response relationships. In contrast, WQS regression, while sensitive in pinpointing important factors, struggles to simultaneously assess joint effects of chemicals with diverse directions of influence (47, 48). In our study, there was a statistically significant negative correlation between the mixed effects of caffeine metabolites and total TT in the male WQS model, while in BKMR, although a negative correlation was also observed, it was not statistically significant. The weight of URXMU7 in WQS exceeds the preset limit, indicating its significant impact on male TT that cannot be ignored. However, no correlation was found between them in the BKMR model. For example, in the female E2 WQS model, no weight of caffeine metabolites exceeding the preset limit was observed, while in the BKMR model, we found URXMU1 and URXMX6 showed positive associations and URXAMU showed a negative association with E2 in females although these relationships are not statistically significant (Supplementary Figure S2). The focus of the results from the WQS and BKMR models is different. To date, has not been directly compared. Using them simultaneously allowed consideration of their strengths and weaknesses, to disentangle the interactions between chemical mixtures.

Caffeine metabolites URXMU2, URXMU6, URXMX3 and URXMX6, are PC2 components in PCA models. Previous studies have shown that the enzyme CYP1A2 in the human liver catalyzes the N-1, N-3 and N-7 demethylations of caffeine to form theobromine, paraxanthine and theophylline, respectively (49). Based on our previous research, PC2 was highly associated with theobromine pathway (44). Theobromine of the xanthine class has been shown to have direct effects on gonadotropin-induced steroidogenesis, mechanistically, theobromine is an inhibitor of phosphodiesterase, adenosine receptor blockers, and histone deacetylase activators (50, 51). A previous study showed that the second and third quartiles of theobromine showed a predicted decrease in mean testosterone compared to the first quartile (37). Thus, caffeine metabolites in PC2 may also act through various pathways to affect sex hormone production and half-life. This explains in some ways the relationships between caffeine metabolites in PC2 (theobromine) and sex hormones, but more researches are needed for the detailed mechanism.

Our analyses have some limitations, First, the main limitation of this research is its cross-sectional design, which hinders clarifying the causal link between urinary caffeine and its metabolites and sex hormones, because caffeine and its metabolites in urine measured in this study could only reflect recent exposures, but changes in sex hormones can be developed over a long time, it might introduce confounding by neglecting the long-term influence of caffeine metabolites. Thus, necessitating additional future studies to confirm the causation nature. Second, caffeine and its metabolites concentrations below the detection limit were replaced with the detection limit divided by the square root of 2, which may result in inaccurate results. Third, we did not perform a weighted analysis on the data. Consequently, the results may not adequately represent the entire US population. However, since we have incorporated factors such as gender, age, and race into the model, the findings of this study can still provide insights into the relationship between caffeine metabolites and sex hormones. Previous research has suggested that when covariates used for calculating sample weights are already part of the regression model, unweighted estimation is favored over weighted estimation (52). However, the applicability of this principle to WQS and BKMR models remains uncertain, besides, WQS and BKMR in R packages do not support data weighting for NHANES data. Given that our analysis aimed to compare various methods and identify a strategy for addressing mixture exposure, our study focused not on conducting a weighted data analysis. Fourth, given that our study’s participants are exclusively Americans, its findings are not broadly relevant to individuals in Asia or elsewhere globally. Nonetheless, the results retain certain reference values as the NHANES cycle includes both non-Hispanic and Asian populations. Fifth, Although BKMR can analyze the influence of interacting compounds on the dependent variable, its focus is not on presenting the interactions themselves, but rather on showcasing the complex, potentially nonlinear, and non-additive effects of each compound on the dependent variable. This was also the focus of our study, hence the results of interactions were not presented.

There were certain advantages to our research. Initially, there’s a significant variation in the biological elements of various coffee varieties, with urinary caffeine and its metabolites serving as reliable indicators of caffeine intake. Consequently, our research mirrored the link between caffeine metabolites and sex hormone risk more precisely than earlier studies. Secondly, this study used three different methods to explore the relationship between caffeine metabolites and sex hormones (multiple linear regression, WQS regression, and BKMR models), these three models evaluated different aspects, delving into the connection between caffeine metabolites and sex hormones through varied viewpoints enriches and broadens the scope of research findings.

To conclude, we applied PCA multiple linear regression, WQS regression, and BKMR regression models to assess the association between caffeine and its metabolites and sex hormones. When considering the results of these three models, we concluded that the whole-body burden of caffeine metabolites, especially the caffeine metabolites in the PC2 metabolic pathway was significantly negatively associated with E2 in males. Our research has proven the importance of applying different methodologies to evaluate the health effects of chemical mixtures. We recommend employing various methods and interpreting their results collectively to arrive at a more reliable conclusion.

## Data Availability

Publicly available datasets were analyzed in this study. This data can be found at: https://wwwn.cdc.gov/nchs/nhanes/default.aspx.
