# Supplementary material for: Associations of urinary caffeine metabolites with sex hormones: comparison of three statistical models
Source: Front Nutr. 2025 Jan 7;11:1497483. doi: 10.3389/fnut.2024.1497483 (PMC11747151; doi:10.3389/fnut.2024.1497483)
Supplement: Supplementary file 1 [file Data_Sheet_1.docx]

Electronic Supporting Materials

**Associations of urinary caffeine metabolites**

**with** **sex hormones: comparison of three statistical models**

Jianli Zhou ^a,†*^, Linyuan Qin ^b, c,†^

^a^ Department of Science and Education, Guilin People’s Hospital, Guilin, 541000, P. R. China.

^b^ Department of Epidemiology and Health Statistics, School of Public Health, Guilin Medical University, Guilin, 541000, P. R. China.

^c^ Guangxi key laboratory of Environmental Exposomics and Entire Lifecycle Health, Guilin, 541000, P. R. China.

*Corresponding author: Jianli Zhou, Department of Science and Education, Guilin People’s Hospital, No. 12 Wenming Road, Guilin, Guangxi, China, Tel: +86-0773-2882100; fax: +86-0773-2881579. Email: [jianliz555@163.com](mailto:jianliz555@163.com).

^†^ These authors contributed equally to this work and share first authorship.

**Supporting Figures**


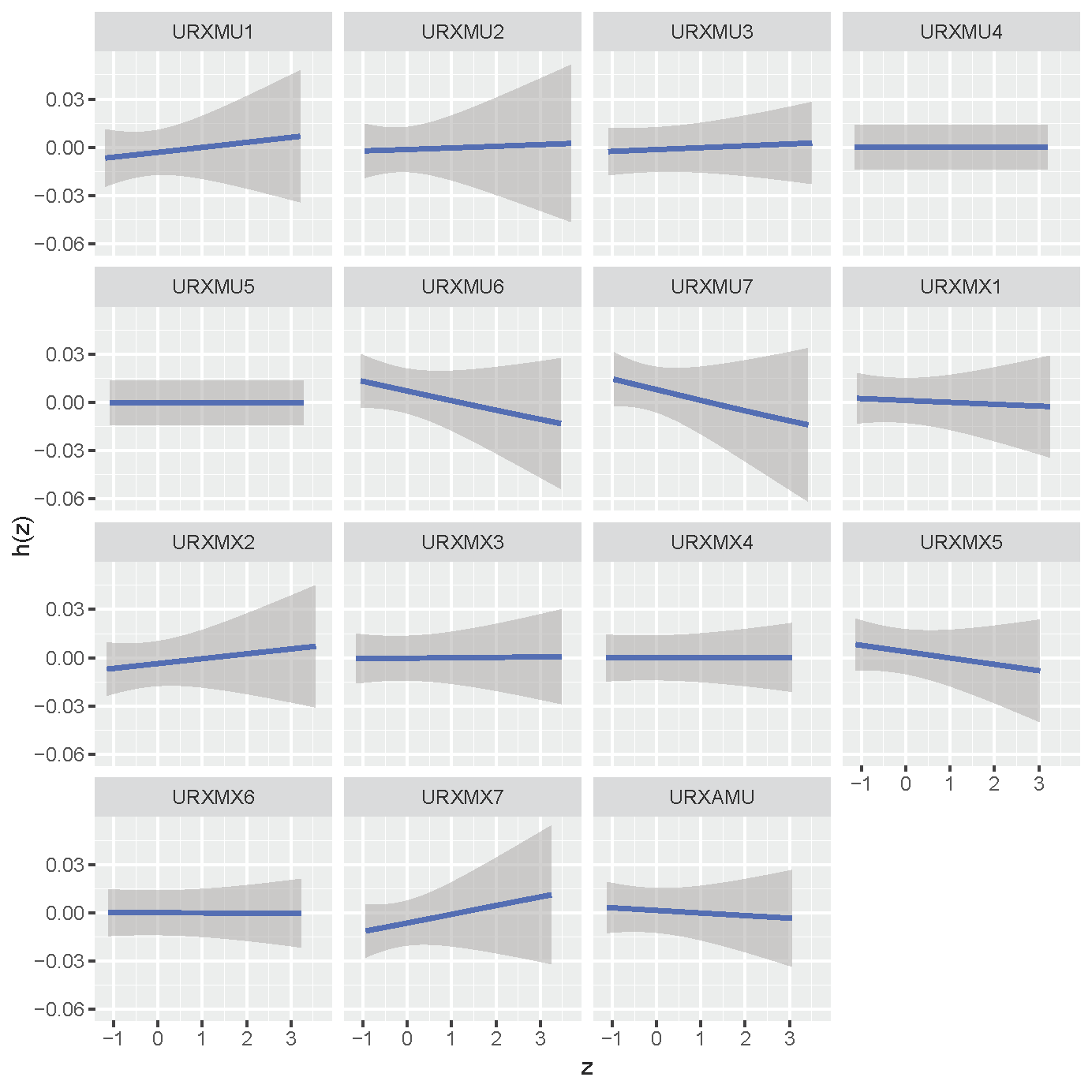


## Figure. S1 Effect of every caffeine metabolites on E2 while fixing other caffeine metabolites at their median level in males. Models were adjusted for gender, age, race, education level, poverty income ratio, marital status, smoking and drinking status, physical activity, BMI, eGFR and daily sleep time. URXMU1, 1-methyluric acid; URXMU2, 3-methyluric acid; URXMU3, 7-methyluric acid; URXMU4, 1,3-dimethyluric acid; URXMU5, 1,7-dimethyluric acid; URXMU6, 3,7-dimethyluric acid; URXMU7, 1,3,7-trimethyluric acid; URXMX1, 1-methylxanthine; URXMX2, 3-methylxanthine; URXMX3, 7-methylxanthine; URXMX4, 1,3-dimethylxanthine, theophylline; URXMX5, 1,7- dimethylxanthine, paraxanthine; URXMX6, 3,7-dimethylxanthine, theobromine; URXMX7, 1,3,7-trimethylxanthine, caffeine; URXAMU, 5-acetylamino-6-amino-3-methyluracil.


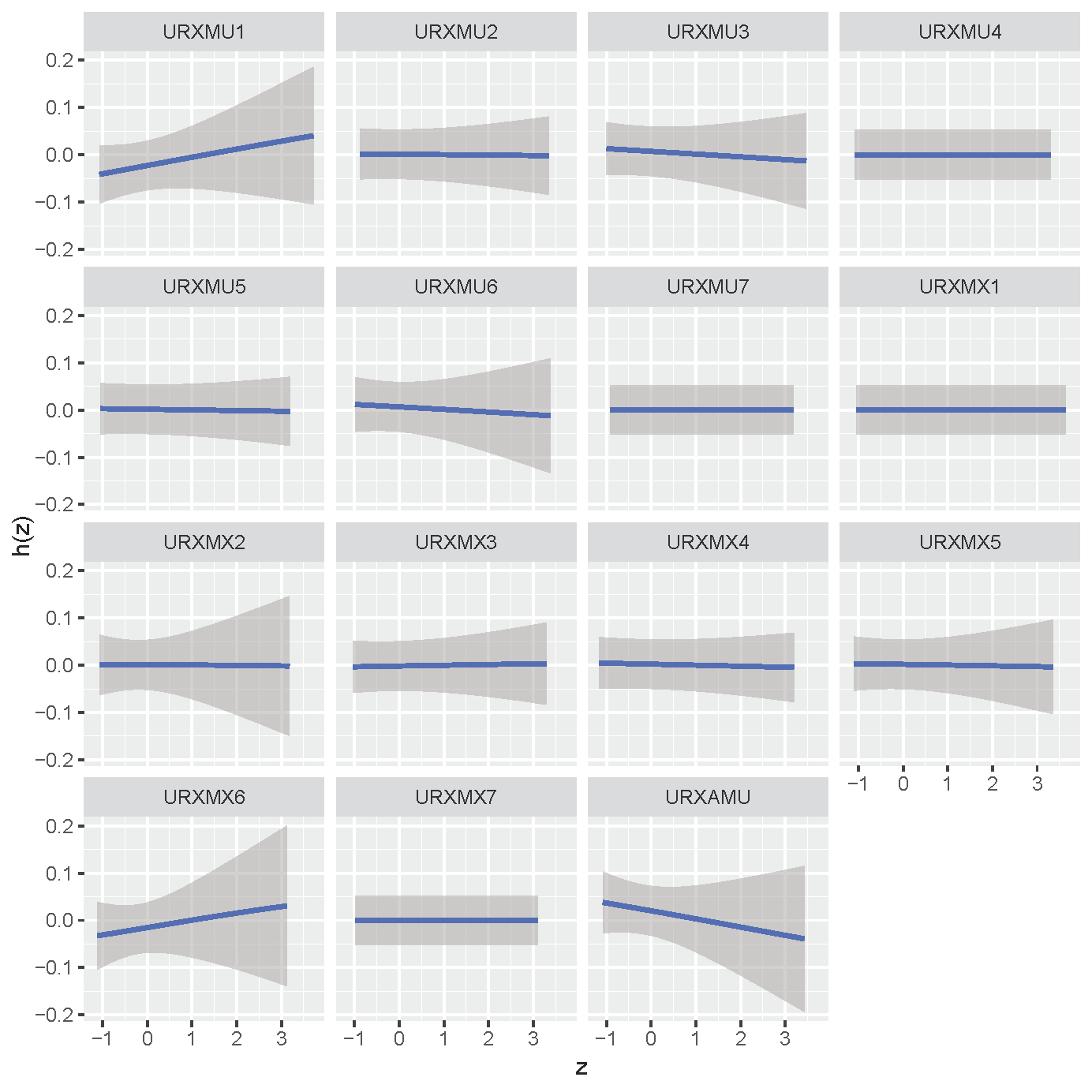


**Figure. S2** Effect of every caffeine metabolites on E2 while fixing other caffeine metabolites at their median level in females. Models were adjusted for gender, age, race, education level, poverty income ratio, marital status, smoking and drinking status, physical activity, BMI, eGFR and daily sleep time. URXMU1, 1-methyluric acid; URXMU2, 3-methyluric acid; URXMU3, 7-methyluric acid; URXMU4, 1,3-dimethyluric acid; URXMU5, 1,7-dimethyluric acid; URXMU6, 3,7-dimethyluric acid; URXMU7, 1,3,7-trimethyluric acid; URXMX1, 1-methylxanthine; URXMX2, 3-methylxanthine; URXMX3, 7-methylxanthine; URXMX4, 1,3-dimethylxanthine, theophylline; URXMX5, 1,7- dimethylxanthine, paraxanthine; URXMX6, 3,7-dimethylxanthine, theobromine; URXMX7, 1,3,7-trimethylxanthine, caffeine; URXAMU, 5-acetylamino-6-amino-3-methyluracil.


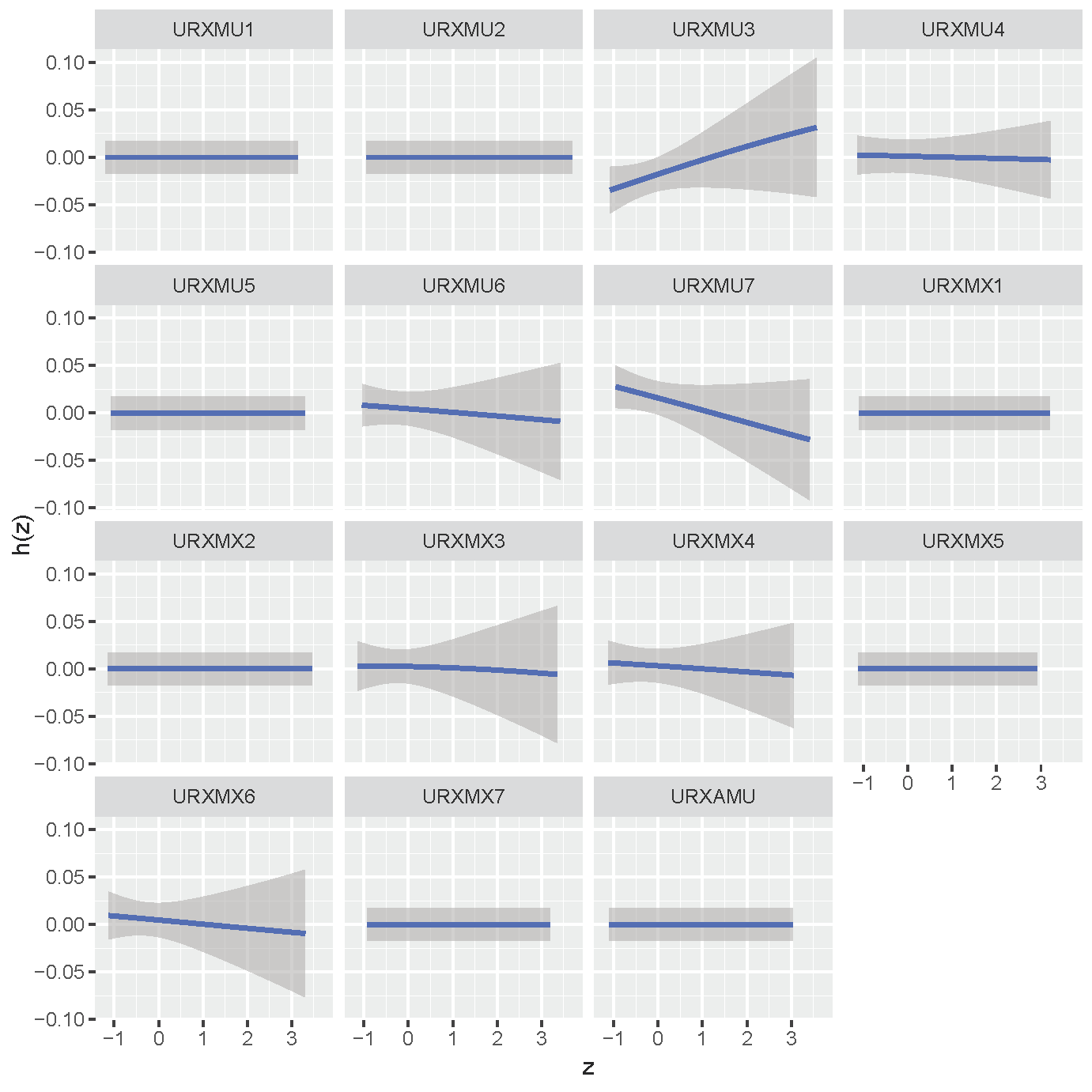


# Figure. S3 Effect of every caffeine metabolites on SHBG while fixing other caffeine metabolites at their median level in males. Models were adjusted for gender, age, race, education level, poverty income ratio, marital status, smoking and drinking status, physical activity, BMI, eGFR and daily sleep time. URXMU1, 1-methyluric acid; URXMU2, 3-methyluric acid; URXMU3, 7-methyluric acid; URXMU4, 1,3-dimethyluric acid; URXMU5, 1,7-dimethyluric acid; URXMU6, 3,7-dimethyluric acid; URXMU7, 1,3,7-trimethyluric acid; URXMX1, 1-methylxanthine; URXMX2, 3-methylxanthine; URXMX3, 7-methylxanthine; URXMX4, 1,3-dimethylxanthine, theophylline; URXMX5, 1,7- dimethylxanthine, paraxanthine; URXMX6, 3,7-dimethylxanthine, theobromine; URXMX7, 1,3,7-trimethylxanthine, caffeine; URXAMU, 5-acetylamino-6-amino-3-methyluracil.


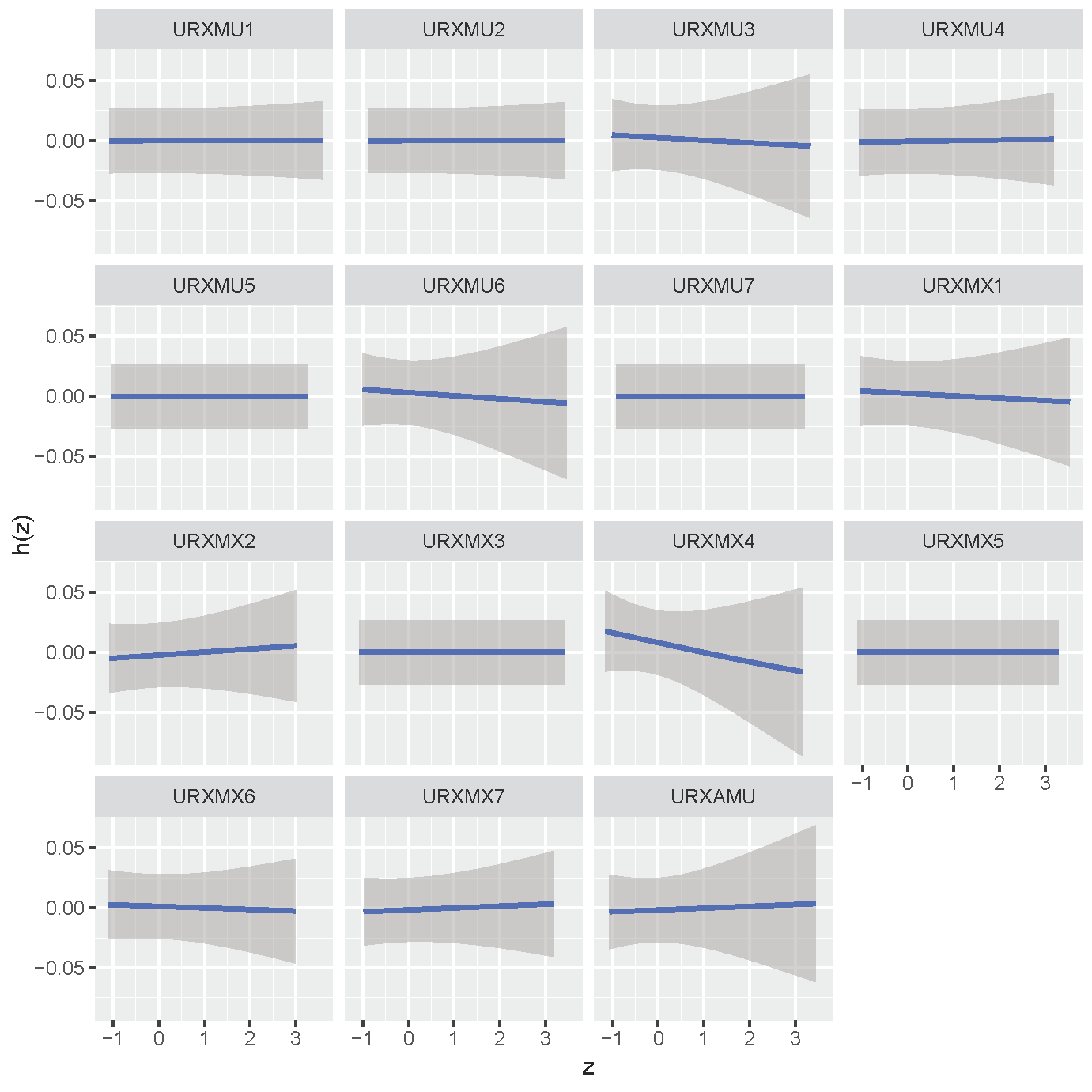


**Figure. S4** Effect of every caffeine metabolites on SHBG while fixing other caffeine metabolites at their median level in females. Models were adjusted for gender, age, race, education level, poverty income ratio, marital status, smoking and drinking status, physical activity, BMI, eGFR and daily sleep time. URXMU1, 1-methyluric acid; URXMU2, 3-methyluric acid; URXMU3, 7-methyluric acid; URXMU4, 1,3-dimethyluric acid; URXMU5, 1,7-dimethyluric acid; URXMU6, 3,7-dimethyluric acid; URXMU7, 1,3,7-trimethyluric acid; URXMX1, 1-methylxanthine; URXMX2, 3-methylxanthine; URXMX3, 7-methylxanthine; URXMX4, 1,3-dimethylxanthine, theophylline; URXMX5, 1,7- dimethylxanthine, paraxanthine; URXMX6, 3,7-dimethylxanthine, theobromine; URXMX7, 1,3,7-trimethylxanthine, caffeine; URXAMU, 5-acetylamino-6-amino-3-methyluracil.


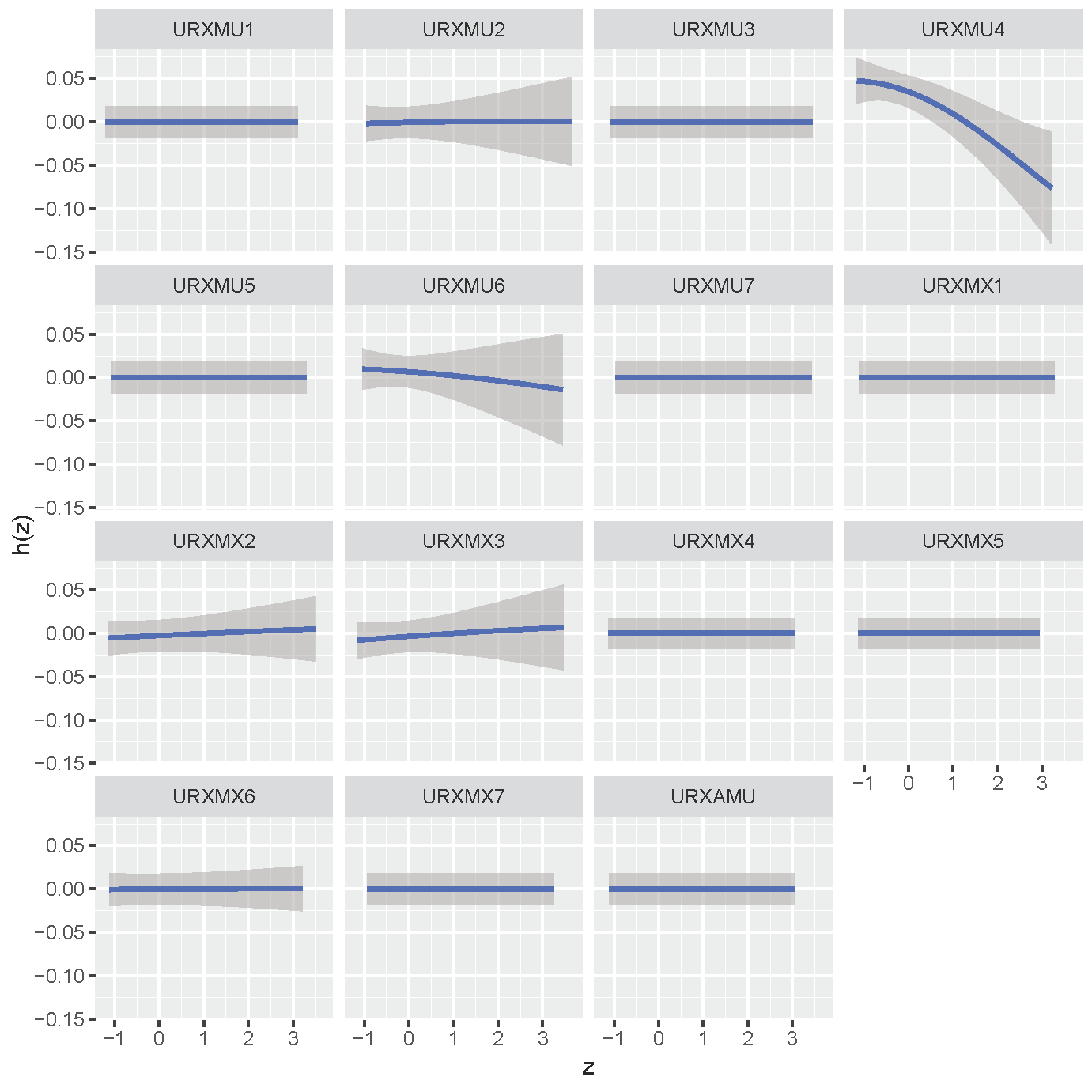


# Figure. S5 Effect of every caffeine metabolites on TT while fixing other caffeine metabolites at their median level in males. Models were adjusted for gender, age, race, education level, poverty income ratio, marital status, smoking and drinking status, physical activity, BMI, eGFR and daily sleep time. URXMU1, 1-methyluric acid; URXMU2, 3-methyluric acid; URXMU3, 7-methyluric acid; URXMU4, 1,3-dimethyluric acid; URXMU5, 1,7-dimethyluric acid; URXMU6, 3,7-dimethyluric acid; URXMU7, 1,3,7-trimethyluric acid; URXMX1, 1-methylxanthine; URXMX2, 3-methylxanthine; URXMX3, 7-methylxanthine; URXMX4, 1,3-dimethylxanthine, theophylline; URXMX5, 1,7- dimethylxanthine, paraxanthine; URXMX6, 3,7-dimethylxanthine, theobromine; URXMX7, 1,3,7-trimethylxanthine, caffeine; URXAMU, 5-acetylamino-6-amino-3-methyluracil.


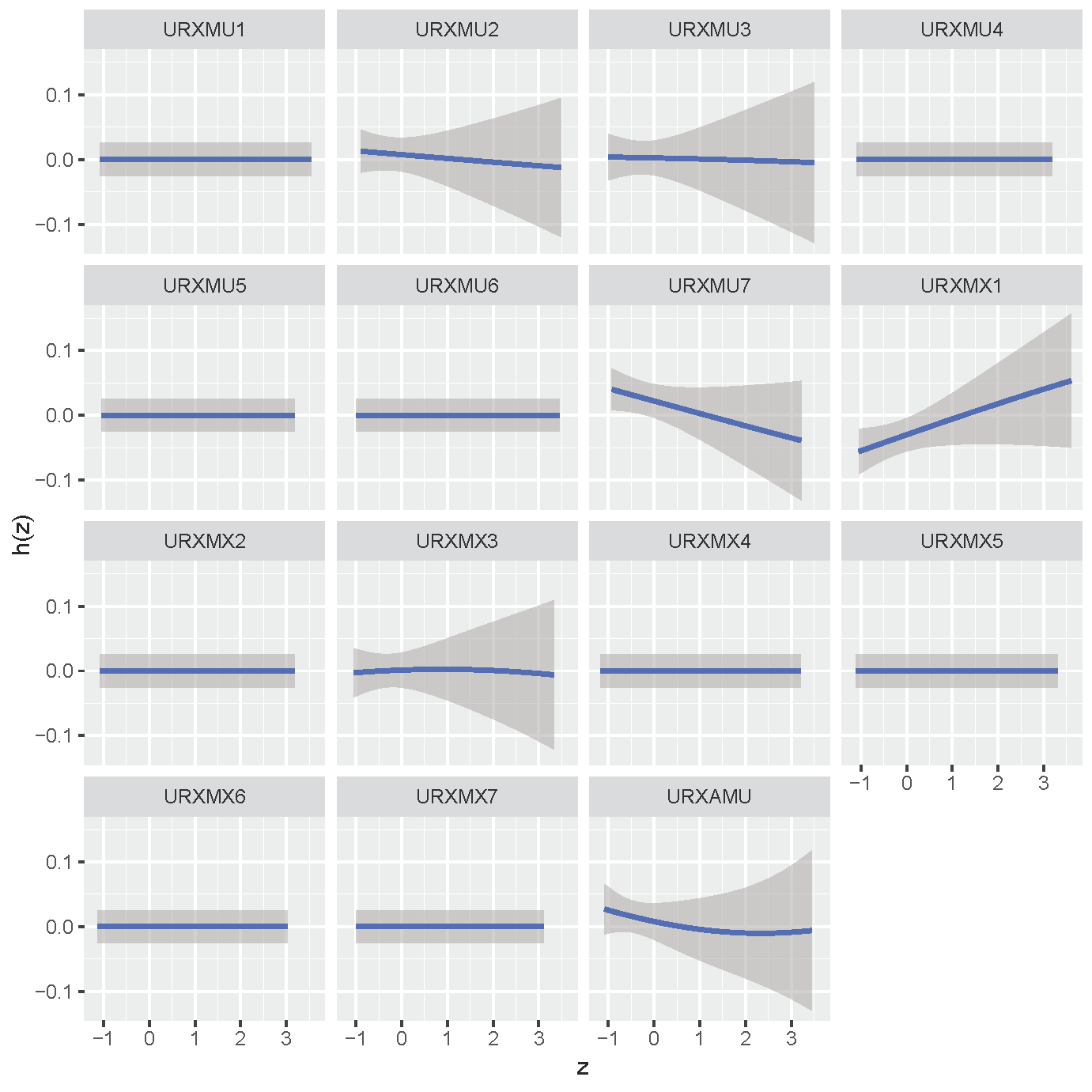


**Figure. S6** Effect of every caffeine metabolites on TT while fixing other caffeine metabolites at their median level in females. Models were adjusted for gender, age, race, education level, poverty income ratio, marital status, smoking and drinking status, physical activity, BMI, eGFR and daily sleep time. URXMU1, 1-methyluric acid; URXMU2, 3-methyluric acid; URXMU3, 7-methyluric acid; URXMU4, 1,3-dimethyluric acid; URXMU5, 1,7-dimethyluric acid; URXMU6, 3,7-dimethyluric acid; URXMU7, 1,3,7-trimethyluric acid; URXMX1, 1-methylxanthine; URXMX2, 3-methylxanthine; URXMX3, 7-methylxanthine; URXMX4, 1,3-dimethylxanthine, theophylline; URXMX5, 1,7- dimethylxanthine, paraxanthine; URXMX6, 3,7-dimethylxanthine, theobromine; URXMX7, 1,3,7-trimethylxanthine, caffeine; URXAMU, 5-acetylamino-6-amino-3-methyluracil.


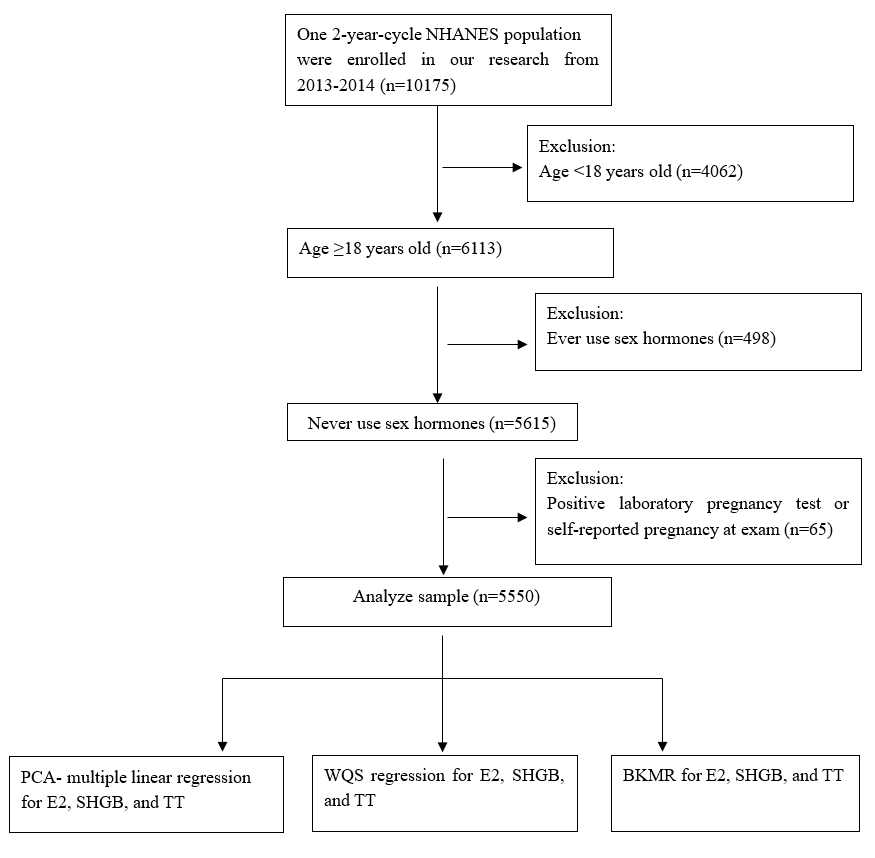


**Figure. S7 Flowchart of population included in our final analysis (N = 5550), NHANES, USA, 2013- -2014.**

**Table S1 The lower limit of detection (LLOD in umol/L) for caffeine and caffeine metabolites:**

| NHANES variable | Caffeine and caffeine metabolites | LLOD |
| --- | --- | --- |
| URXMU1 | 1-methyluric acid (umol/L) | 0.05 |
| URXMU2 | 3-methyluric acid (umol/L) | 0.1 |
| URXMU3 | 7-methyluric acid (umol/L) | 0.04 |
| URXMU4 | 1,3-dimethyluric acid (umol/L) | 0.02 |
| URXMU5 | 1,7-dimethyluric acid (umol/L) | 0.02 |
| URXMU6 | 3,7-dimethyluric acid (umol/L) | 0.03 |
| URXMU7 | 1,3,7-trimethyluric acid (umol/L) | 0.005 |
| URXMX1 | 1-methylxanthine (umol/L) | 0.03 |
| URXMX2 | 3-methylxanthine (umol/L) | 0.04 |
| URXMX3 | 7-methylxanthine (umol/L) | 0.02 |
| URXMX4 | 1,3-dimethylxanthine (theophylline) (umol/L) | 0.01 |
| URXMX5 | 1,7-dimethylxanthine (paraxanthine) (umol/L) | 0.006 |
| URXMX6 | 3,7-dimethylxanthine (theobromine) (umol/L) | 0.004 |
| URXMX7 | 1,3,7-trimethylxanthine (caffeine) (umol/L) | 0.003 |
| URXAMU | 5-acetylamino-6-amino-3-methyluracil | 0.1 |
